# A network analysis of obsessive-compulsive patients in intensive outpatient treatment

**DOI:** 10.1192/j.eurpsy.2026.10184

**Published:** 2026-03-23

**Authors:** Valerie S. Swisher, Kate Rogers, Sandra Hadlock, Michelle G. Newman

**Affiliations:** 1 The Pennsylvania State University, USA; 2 OCD Anxiety Centers, USA

**Keywords:** ERP, network analysis, obsessive-compulsive disorder, treatment response, intensive outpatient treatment

## Abstract

**Background:**

The network theory of mental disorders posits that associations between symptoms activate other symptoms to maintain a disorder over time. Network analytic approaches therefore may inform treatment targets. In the present study, we compared baseline OCD symptom networks among treatment responders to non-responders and examined how network structure and connectivity changed from before to after exposure and response prevention (ERP) treatment.

**Methods:**

Community adults with OCD (*n* = 712) who underwent intensive outpatient treatment were assessed using the Yale-Brown Obsessive Compulsive Scale (YBOCS) at admission and discharge. Network comparison tests were used to (a) examine differences in baseline symptom network structures between treatment responders versus non-responders and (b) examine changes in network structures from pre- to post-treatment.

**Results:**

Pre-treatment network structures and global connectivity did not differ significantly between treatment responders and non-responders. However, post-treatment networks exhibited greater global strength (i.e., stronger associations between OCD symptoms) and significantly different network structure (i.e., different patterns of associations between OCD symptoms) relative to the pre-treatment network.

**Conclusions:**

Findings showed that network structure and connectivity in OCD may be more informative as a marker of therapeutic change than in discriminating treatment responders from nonresponders using baseline symptoms. After ERP treatment, associations between obsessions and compulsions demonstrated significantly greater global network strength and altered network structure, thus underscoring the potential for network approaches to identify mechanisms of change throughout OCD treatment. Future studies incorporating session-by-session data may clarify when and how these network shifts occur over the course of therapy to help identify treatment targets.

## Introduction

Understanding how psychological symptoms relate to and reinforce one another is a useful framework that may inform novel treatment targets. Such is the foundation of network theory of psychopathology, which posits that individual symptoms are interrelated and causally influence one another, rather than being the byproduct of a latent disease [1, 2]. Derived from this theory, network analysis is a statistical method that allows researchers to examine how individual symptoms relate to and maintain all other symptoms in the network. Such analyses can be helpful to elucidate potential symptoms that are maintaining or exacerbating psychopathological processes, which may help identify potential intervention targets.

This conceptual framework might be especially relevant to obsessive-compulsive disorder (OCD), where a reinforcement cycle of obsessions (resulting in anxiety) and compulsions (resulting in relief) is thought to maintain the condition [3, 4]. OCD is a chronic, often debilitating condition whereby individuals experience obsessions (i.e., intrusive, unwanted thoughts) and compulsions (i.e., repetitive, compensatory behaviors in response to the obsession) for at least 1 hour per day [5]. It creates substantial functional impairment [6], with individuals often requiring more intensive care, including intensive outpatient programming (IOP) or residential/in-patient care [7].

Symptom-based networks of OCD may reveal important findings regarding how individual symptoms of OCD co-activate one another. However, most studies using network analysis in OCD examined how comorbidities [8–13] or relevant associated mechanisms, such as obsessive beliefs [14], related to OCD symptoms. Although informative, these analyses do not reveal whether baseline OCD symptom networks can discriminate treatment responders from nonresponders and how individual OCD symptom co-activation changes as a result of treatment. This is important because network theory posits that the individual symptoms of OCD are interrelated and causally influence each other’s severity to maintain the diagnosis. The theory further suggests that there may be key OCD symptoms that drive the maintenance of other OCD symptoms, and these key symptoms may be important to target during treatment. Understanding pre-treatment differences in OCD symptom networks and how networks change from pre- to post-treatment without other variables in the network is important since the primary goal of exposure and response prevention (ERP) is to treat OCD symptoms. Thus, studying networks of individual OCD symptoms could help identify potential treatment targets at the OCD symptom level. Given that exposure and response prevention (ERP) directly targets OCD symptoms, examining longitudinal changes in symptom-to-symptom relationships also provides a more proximal test of how ERP exerts its therapeutic effects on OCD. However, only two known studies have examined how OCD symptoms relate to one another in the context of treatment.

In a large sample of patients with OCD who underwent exposure and response prevention (ERP) in an intensive outpatient setting (IOP), Kuckertz et al. [15] examined how baseline network structure (patterns of association between symptoms) and connectivity (strength of associations between symptoms) differed between treatment responders relative to non-responders based on the 10-items from the Yale Brown Obsessive-Compulsive Scale (YBOCS). Non-responders had a stronger association between distress associated with compulsions and both difficulty controlling compulsions and interference from compulsions. Furthermore, non-responders had a weaker association between distress associated with obsessions and distress associated with compulsions. The authors concluded that non-responders may exhibit greater difficulty tolerating distress associated with compulsions relative to responders. Although informative, these findings only examined OCD symptoms at pre-treatment and did not examine changes in network structure and connectivity after participants completed treatment. This limits our understanding of how network structures and edge-to-edge associations (i.e., partial correlations among individual symptoms) change as participants undergo the treatment process. Moreover, although the authors examined which symptoms had the highest strength centrality in the responder and non-responder networks, they did not examine whether these symptoms differed significantly in strength centrality from other symptoms within the two networks. As such, it was unclear whether symptoms with the strongest strength centrality in responders (versus nonresponders) were meaningfully different than other symptoms. This is important, as identifying symptoms that have significantly greater strength centrality in the network may be informative in identifying potential treatment targets.

Recent work by Kim et al. [16] expanded on these findings by using the 10-item YBOCS symptom severity scale to examine changes in OCD symptom networks over the course of outpatient ERP treatment across three progress trajectories (dramatic progress, moderate progress, and little to no progress). Unlike Kuckertz et al. [15], this study used a Pearson correlation-based network analysis to estimate marginal associations between symptoms, rather than regularized partial correlation networks and examined network modularity (i.e., the degree to which the network was divided into non-overlapping subgroups) [17]. Results showed that networks clustered into two distinct modules across progress trajectories, one related to resistance/control due to obsessions and compulsions and the other related to interference/distress due to obsessions and compulsions. Additionally, these modules became more integrated (i.e., individual symptoms (nodes) and more strongly and densely connected to one another, rather than clustering into modules) over the course of treatment for the dramatic and moderate progress classes, but not for the little-to-no progress class. Thus, overall, findings showed that successful ERP resulted in a more integrated, densely connected network, rather than being indicative of greater symptom activation and psychological vulnerability, as network theory suggests [1, 2]. The authors also suggested their findings revealed a clinically meaningful feedback loop, whereby greater control/resistance of obsessions and compulsions led to less interference/distress from obsessions and compulsions and vice versa. However, because of their use of Pearson, rather than partial, correlations, it was unclear how unique relationships between symptoms changed over the course of treatment—specifically, which symptoms exerted the greatest influence on the broader network when controlling for other associations. As such, the extent to which individual symptoms differed in strength centrality (i.e., their overall connectedness to other symptoms) or whether particular edge-to-edge associations (connections between specific symptom pairs) meaningfully shifted with treatment progress remained unknown. Identifying these unique and central symptom relationships is important, as it may reveal key mechanisms of change in ERP—highlighting which symptoms are most instrumental in driving broader symptom improvement and offering insight into potential targets for optimizing treatment outcomes.

Although few studies have examined OCD symptom networks in the context of treatment, research on other forms of psychopathology offers important insights. Network theory suggests that as symptoms of a particular disorder decrease in severity and frequency during treatment, the network should become less dense (global connectivity should decrease)—indicating weaker associations among symptoms [18, 19]. The theory also suggests that effective treatment will reduce the level of connectivity between key nodes, breaking their causal links (network structure) [18, 19]. However, empirical evidence has not supported this prediction. In studies of posttraumatic stress disorder (PTSD), generalized anxiety disorder (GAD), and major depressive disorder (MDD), whereas symptom severity significantly decreased from pre- to post-treatment, network structures remained largely stable, and global connectivity increased [20–23]. Several explanations have been proposed. Some symptom relationships were theorized to be biologically ingrained (e.g., nightmares and poor sleep in PTSD) and therefore resistant to therapeutic change [21]. Alternatively, this may have been due to less variability between patients at pre-treatment (because they were selected based on extreme scores) and greater variability at post-treatment (due to differential treatment response), which could have increased correlation coefficients and thereby increased global network strength. Nonetheless, findings question the assumption that higher density networks reflect greater symptom severity. Should the current study replicate prior study findings, the theory may need to be revised to more accurately explain symptom dynamics and recovery pathways over the course of treatment.

In sum, although existing network-based studies have provided valuable insight into how OCD symptoms interrelate, it remains unclear how these relationships differ before and after treatment, and which specific symptoms or connections are most central to recovery. Previous work has either examined networks at a single time point [15] or relied on correlation-based methods that did not account for unique associations among symptoms [16]. Consequently, it is still unknown whether the structure, connectivity, or centrality of baseline OCD symptom networks can significantly discriminate responders from nonresponders and whether such networks meaningfully change following ERP when accounting for shared variance among symptoms. Moreover, findings from research on other psychopathologies have challenged the assumption that symptom networks become less dense as symptoms remit, highlighting the need to test whether this theoretical pattern holds true for OCD. Understanding whether global network strength (association between symptoms) decreases, remains stable, or even increases after treatment could offer insight into how recovery unfolds at the symptom level. Furthermore, post-treatment change in network structure could be an informative approach to examining treatment targets.

Therefore, the present study had two primary aims. Our first aim was to examine differences in pre-treatment symptom networks between treatment responders versus non-responders in terms of network invariance (i.e., the extent to which network structure or patterns of association between OCD symptoms were similar across groups) and global network strength (i.e., the extent to which the overall connectivity or strength of association between OCD symptoms was similar across groups). Where significant differences in network structure were found, we also examined whether there were differences in specific edge-to-edge associations. We also aimed to determine the symptoms with the greatest strength centrality in each network. Based on findings by Kuckertz et al. [15], we expected to see significant differences between responders and non-responders in network structure, but not global strength. As significant differences in strength centrality were not examined in prior work [15], it was exploratory whether or not specific symptoms would significantly differ in strength centrality within each network. The second aim was to determine if OCD symptom networks changed from pre- to post-treatment. Specifically, we compared network invariance and global network strength of the pre- and post-treatment networks in the full sample. Based on prior findings, we hypothesized that OCD symptom networks would increase in global network strength but remain unchanged in structure from pre- to post-treatment. Contingent on change being evidenced in the full sample, we also aimed to examine change in pre- to post-treatment networks among responders and non-responders. Based on Kim et al. [16], we expected networks to become significantly more integrated (i.e., individual symptoms (nodes) to become more strongly and densely connected to one another) for the responder but not the non-responder network from pre- to post-treatment. It was exploratory whether network structure would differ in pre-to-post treatment networks for responders and non-responders.

## Method

### Participants

Participants were 712 patients (59.9% female) aged 18–72 years (*M* = 26.87, *SD* = 9.17) who received intensive outpatient ERP (IOP) at the OCD Anxiety Centers^1^ between February 2021 and September 2025 (see Table 1 for demographic and clinical characteristics). All participants provided written informed consent at the onset of treatment for de-identified data to be used in archival research. Participant data were only included in the present study if participants had a primary diagnosis of OCD made by the assigned therapist at the time of intake. Almost half (*n* = 349; 49.0%) of participants did not have a reported co-occurring psychiatric condition. The most common comorbid diagnoses were social anxiety disorder (*n* = 144; *n* = 20.2%), GAD (*n* = 82, 11.5%), PTSD (*n* = 75; 10.5%), or MDD (*n* = 69, 9.7%). However, semi-structured diagnostic interviews were not systematically administered across participating outpatient clinics; therefore, comorbid diagnoses were extracted from routine clinical intake documentation, which varied in structure and diagnostic specificity across sites.

**Table 1.** Participant characteristics of responders and non-responders

	Group differences	Full sample *N* = 712 M (SD) / *n* (%)	Responders *N* = 446 M (SD) / *n* (%)	Non-responders *N* = 266 M (SD) / *n* (%)
Age	*t*(524.94) = 0.93 *p* = 0.349 *d* = 0.08	26.87 (9.17)	26.61 (8.91)	27.30 (9.60)
Gender^a^	*X*^2^ (1) = 0.17 *p* = 0.683	Female: 425 (59.7%) Male: 283 (39.7%) Unknown: 4 (0.6%)	Female: 269 (60.3%) Male: 174 (39.0%) Unknown: 3 (0.7%)	Female: 156 (58.6%) Male: 109 (41.0%) Unknown: 1 (0.4%)
Length of stay (weeks)^*^	*t*(515.49) = −5.96 *p* < .001 *d* = −0.52	11.11 (4.57)	11.89 (4.29)	9.79 (4.72)
YBOCS Pre-Treatment^*^	*t*(516.31) = 3.75 *p* < .001 *d* = 0.33	23.81 (5.84)	24.46 (5.57)	22.74 (6.12)
YBOCS Post-Treatment^*^	*t*(509.17) = −30.92 *p* < .001 *d* = −2.74	13.14 (7.56)	8.67 (4.65)	20.64 (5.19)

*Note*: *p < .001.

Abbreviation: YBOCS = Yale-Brown Obsessive-Compulsive Scale Self-Report.

a For chi-square test, gender was assessed as a binary variable (female/non-female).

### Measures

#### Yale-Brown Obsessive-Compulsive Scale Self-Report (YBOCS-SR)

The YBOCS-SR is a 10-item self-report measure of OCD severity[24]. It assesses time spent, distress, interference, resistance, and control over obsessions (5 items) and compulsions (5 items) in the past week. Items are rated on a 0–4 Likert scale, with higher scores indicating greater symptom severity. Items can be summed to derive a severity score, an obsession subscale score, and a compulsion subscale score. Total scores of ≥16 are considered clinically significant OCD symptoms [25]. It exhibits strong convergent validity with the YBOCS clinician-administered version [24], good internal consistency [26], and sensitivity to treatment changes [27]. Internal consistency in the present sample was good at pre- (*α* = 0.86) and post-treatment (*α* = 0.96).

### Treatment

IOP consisted of 3-hour daily treatment, 5 days per week, with the length of stay in the program tailored to individual client needs. On average, participants stayed in treatment for approximately 11 weeks (*M* = 11.11; *SD* = 4.57). Therapists were master’s or doctoral level psychologists, interns, mental health counselors, clinical social workers, and marriage and family therapists. Therapists received structured, in-house training in ERP for OCD, the primary treatment approach. Participants completed the pre-treatment YBOCS within 3 days of admission and once per week (at the end of the week) for the duration of treatment. Fidelity was monitored via weekly team meetings, in which all therapists received supervision and support from more experienced clinicians. Both telehealth and in-person options were offered to clients, with the majority (82.7%) choosing in-person therapy.

### Data analysis

All analyses were conducted in R Studio, Version 4.4.2. All item-level data were included in analyses. In addition, there were no missing data as YBOCS scores from the last treatment session attended were used as the post-treatment measure.

#### Network estimation

Partial correlation networks were estimated using the *qgraph* package that applies the EBICglasso and qgraph functions [28, 29]. The EBICglasso function regularizes the network with a graphical least absolute shrinkage operator (LASSO) algorithm based on the Extended Bayesian Information Criterion (EBIC) hyperparameter (default of gamma = 0.5), which applies L1 regularization to remove small partial correlations and retain only those with the largest magnitude, removing likely spurious associations [29].

#### Centrality

The strength centrality of each symptom was calculated by summing the absolute edge weights of this symptom, using the qgraph function. Strength centrality indicates how strongly each symptom is associated with all other symptoms. Betweenness and closeness were not used as these parameters are less stable [20, 29].

#### Edge weight accuracy and network stability

To determine edge weight accuracy of the network (i.e., the extent to which the edge weights remained consistent after resampling), we used non-parametric bootstrapped 95% confidence intervals of estimated edge weights with 10,000 samples [29]. To determine the stability of the strength centrality indices, we calculated the Centrality Stability (CS) coefficient by using the case-dropping subset bootstrap method with 10,000 samples, which determines if the order of strength centrality indices remains the same as an increasing percentage of participants sampled are dropped. Epskamp and colleagues [29] suggested that CS coefficient values below 0.25 indicate that centrality indices should not be interpreted and that values above 0.5 are preferable.

#### Aim 1: Compare pre-treatment symptom networks of responders to non-responders

Participants were categorized as treatment responders if they achieved 35% or greater YBOCS total score reduction from pre-treatment to post-treatment. This cutoff was based on prior guidelines [30, 31] and used by Kuckertz et al. [15]. It resulted in roughly equal group sizes to create two separate partial correlation networks for treatment responders and non-responders at pre-treatment. The NetworkComparisonTest (NCT) package [32] was used, set at 1000 iterations. Pre-treatment responder and non-responder networks were compared on network invariance (*M*; i.e., the extent to which network structure was similar across groups) and network global strength (*S*; i.e., the extent to which the overall connectivity between nodes was similar across groups). Where differences in network structure were found, edge invariance tests were used to determine which specific symptom associations were (i.e., edges) different between networks.

#### Aim 2: Examine changes in pre- to post- treatment symptom networks in the full sample and among treatment responders and non-responders

To determine changes in global network strength and network structure from pre-treatment to post-treatment, the NCT package was also used [32], with parameters set to account for repeated measurement. As we aimed to examine overall network changes from treatment, the full sample was used (i.e., both responders and non-responders, *n =* 712). Contingent on network differences in the full sample being found, the same analysis was also conducted to examine changes in global network strength and network invariance from pre-treatment to post-treatment separately among responders and non-responders. Lastly, as in Aim 1, where network structure differences were found, edge invariance tests were used to determine which specific symptom associations (i.e., edges) differed between networks.

## Results

### Aim 1: Compare pre-treatment symptom networks of responders to non-responders

#### Responders and non-responders

The majority (*n* = 446; 62.6%) of participants were classified as responders and 37.3% (*n* = 266) were considered non-responders. There were no significant differences in age or sex between responders and non-responders, *ps* = .350–.683. Responders had significantly greater pre-treatment YBOCS total scores (*p* < .001, *d* = 0.33) and length of stay (in weeks) (*p* < .001, *d* = 0.52), with a 1.82-point higher mean YBOCS score at pre-treatment and remained in treatment for approximately 14.8 days longer than non-responders. Non-responders had significantly higher post-treatment YBOCS total scores than non-responders, *p* < .001, with a large effect size, *d* = −2.74. See Table 1 for all demographic and participant characteristics and group comparisons.

#### Network stability, structure, and strength centrality of pre-treatment responder and non-responder networks

**Figure 1.**
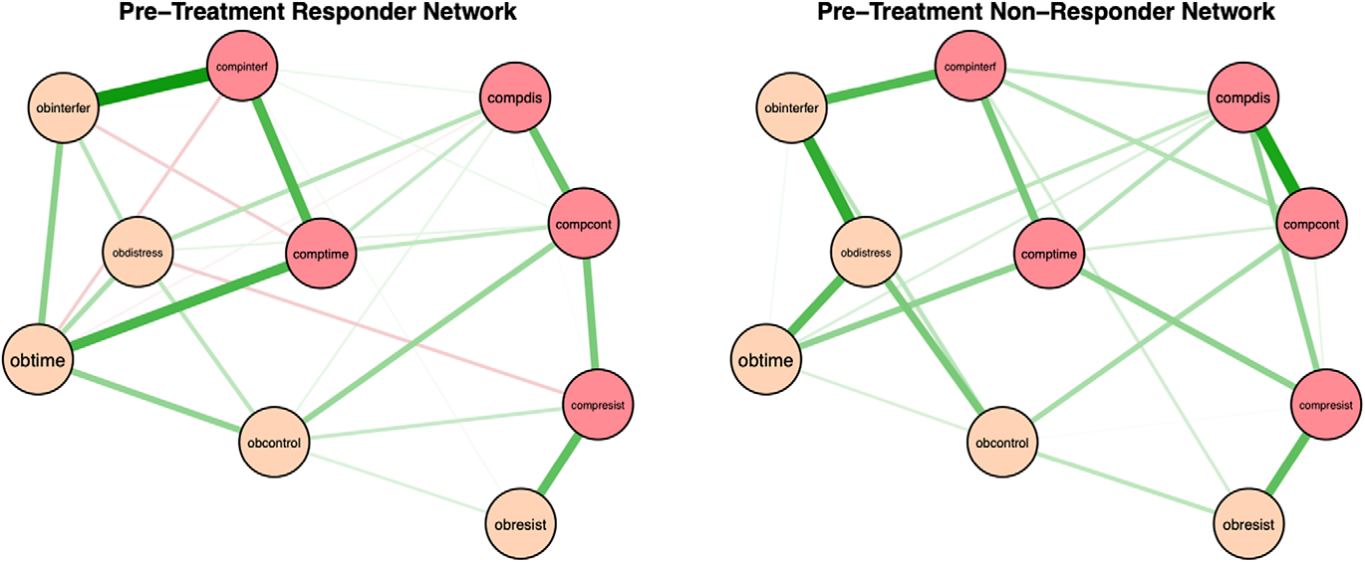
Regularized partial correlation networks for responders and non-responders at pre treatment. *Note*: Responders (i.e., participants who experienced ≥ 35% reduction in YBOCS scores from pre- to post-treatment) at pre-treatment are presented on the left. Non-responders at pre-treatment are on the right. Positive correlations are represented in green and negative correlations in red, with thicker lines representing stronger partial correlations. Obtime = time consumed by obsessions; Obinterfer = interference due to obsessions; Obdistress = distress caused by obsessions, Obresist = difficulty resisting obsessions; Obcontrol = difficulty controlling obsessions; Comptime = time consumed by compulsions; Compinterf = interference due to compulsions; Compdis = distress caused by compulsions; Compresis = difficulty resisting compulsions; Compcont = difficulty controlling compulsions.

**Figure 2.**
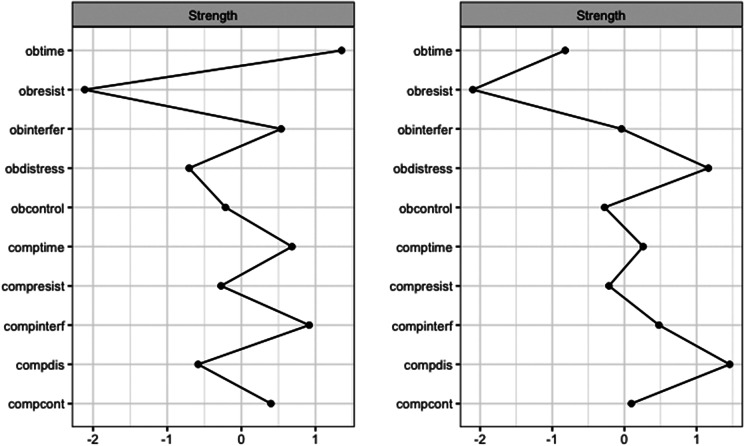
Strength Centrality Plot for Responders and Non-Responders at Pre-Treatment. *Note*: Strength centrality plots for responders (left) and non-responders (right) at pre-treatment. Nodes are presented on the y-axis and z-scores on the x-axis. Obtime = time consumed by obsessions; Obinterfer = interference due to obsessions; Obdistress = distress caused by obsessions, Obresist = difficulty resisting obsessions; Obcontrol = difficulty controlling obsessions; Comptime = time consumed by compulsions; Compinterf = interference due to compulsions; Compdis = distress caused by compulsions; Compresis = difficulty resisting compulsions; Compcont = difficulty controlling compulsions.

Pre-treatment network structures and strength centrality indices for responders and non-responders are shown in Figures 1 and 2. Bootstrapped 95% confidence intervals for edge weights for responder and non-responder networks at pre- and post-treatment are presented in Supplementary Figure S1. Most edge weights showed limited overlap in confidence intervals, indicating that edge estimates were stable and interpretable. Correlation stability coefficients (.59 and .44 for responders and non-responders, respectively; see Supplementary Figure S2) suggest that strength centrality indices were interpretable for both pre-treatment responder and non-responder networks. For responders at pre-treatment, time spent performing compulsions, time spent obsessing, and interference due to compulsions had the highest strength centrality, although they did not significantly differ in strength centrality from most other nodes. Difficulty resisting obsessions significantly differed from all other nodes in strength centrality, such that it had significantly less strength centrality than all other nodes (see Supplementary Figure S3). For non-responders at pre-treatment, distress associated with compulsions, distress associated with obsessions, and interference due to compulsions had the highest strength centrality, but also did not significantly differ in strength centrality from most other nodes. Difficulty resisting obsessions also significantly differed from all other nodes in strength centrality in the non-responder network, exhibiting significantly less strength centrality than all other nodes (see Supplementary Figure S3).

#### Comparison between pre-treatment responder and non-responder networks

Between-group network comparison tests revealed no significant differences in network invariance (*M* = 0.20; *p* = .068) and global network strength (*S* = 0.06; *p* = .814). As no structural or connectivity differences were found, edge differences between responders and non-responders were not examined [32].

### Aim 2: Examine changes in pre- to post-treatment symptom networks in the full sample and in treatment responders and non-responders

#### Network structure, strength centrality, edge weight accuracy, and stability

**Figure 3.**
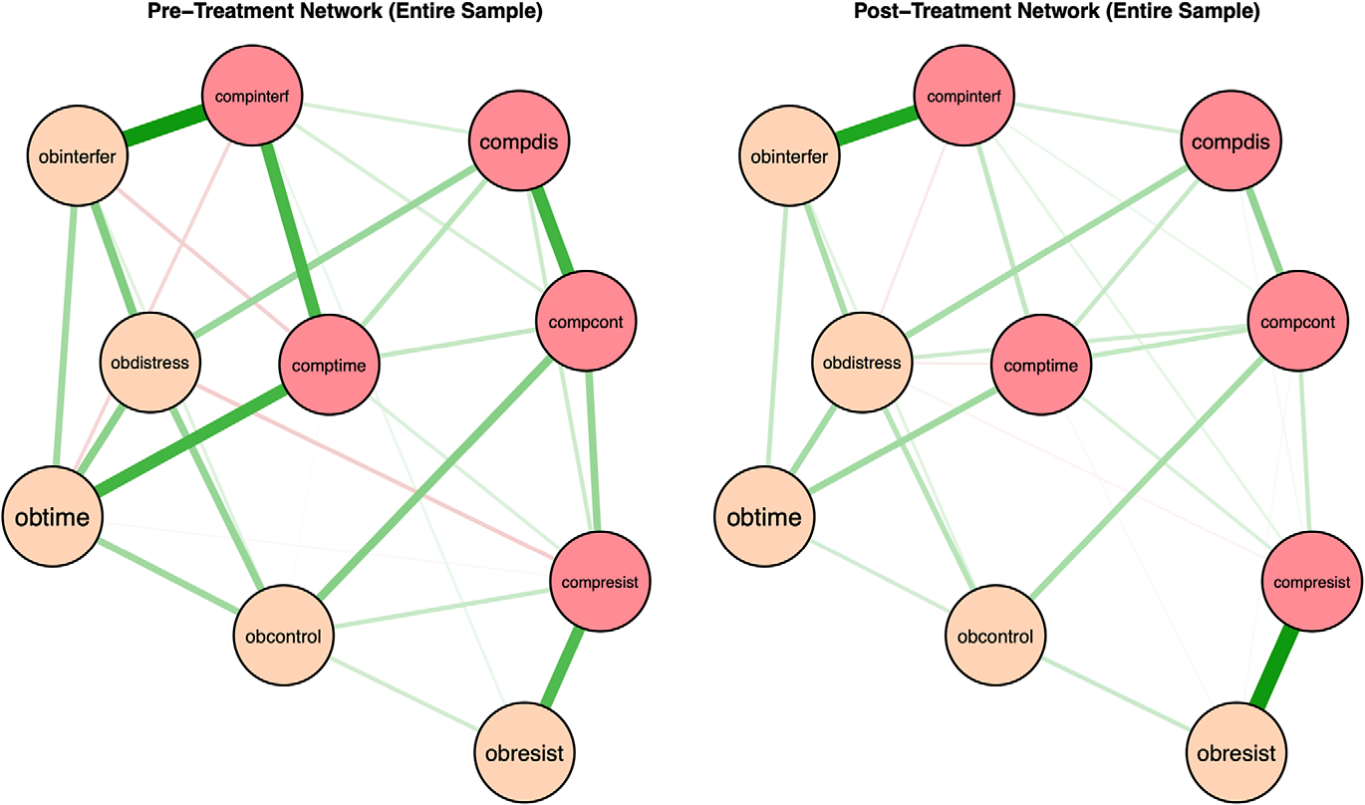
Regularized partial correlation networks for the entire sample at pre- and post-treatment. *Note*: Positive correlations are represented in green and negative correlations are presented in red, with thicker lines representing stronger partial correlations. Obtime = time consumed by obsessions; Obinterfer = interference due to obsessions; Obdistress = distress caused by obsessions, Obresist = difficulty resisting obsessions; Obcontrol = difficulty controlling obsessions; Comptime = time consumed by compulsions; Compinterf = interference due to compulsions; Compdis = distress caused by compulsions; Compresist = difficulty resisting compulsions; Compcont = difficulty controlling compulsions.

**Figure 4.**
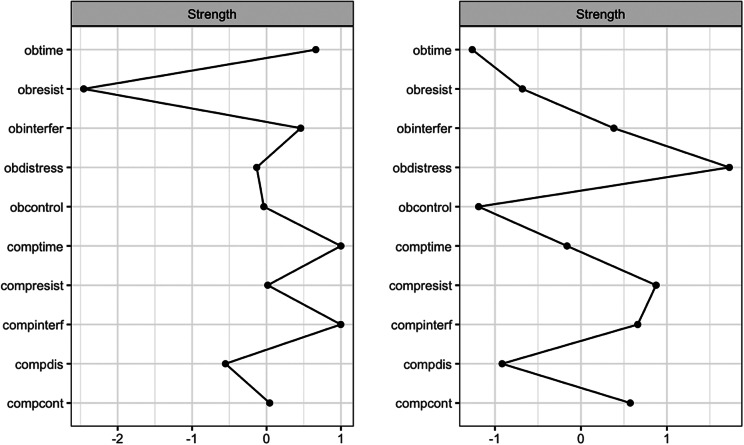
Strength Centrality Plot at Pre- and Post-Treatment for the Entire Sample. *Note*: Strength centrality plots at pre-treatment (left) and post-treatment (right) for the entire sample are presented. Nodes are presented on the y-axis and z-scores on the x-axis. Obtime = time consumed by obsessions; Obinterfer = interference due to obsessions; Obdistress = distress caused by obsessions, Obresist = difficulty resisting obsessions; Obcontrol = difficulty controlling obsessions; Comptime = time consumed by compulsions; Compinterf = interference due to compulsions; Compdis = distress caused by compulsions; Compresis = difficulty resisting compulsions; Compcont = difficulty controlling compulsions.

Network structures and strength centrality indices for the full sample at pre- and post- treatment are shown in Figures 3 and 4. Bootstrapped 95% confidence intervals for edge weights for responder and non-responder networks at pre- and post-treatment are presented in Supplementary Figure S4, indicating that edge estimates were stable and interpretable. Correlation stability coefficients were strong at pre-treatment (0.59) and acceptable at post-treatment (0.28; see Supplementary Figure S5). At pre-treatment, time spent performing compulsions, interference due to compulsions, and time spent obsessing showed the highest strength centrality. Resistance to obsessions significantly differed from all other nodes in strength centrality, and distress from obsessions significantly differed from difficulty controlling obsessions in strength centrality (see Supplementary Figure S6). For the post-treatment network, distress due to obsessions, resistance to compulsions, and interference due to compulsions had the greatest strength centrality. Only resistance to obsessions significantly differed from most other nodes in strength centrality, except for time spent obsessing and difficulty controlling obsessions (Supplementary Figure S6).

#### Comparison between pre-treatment and post-treatment networks in the full sample and in treatment responders and non-responders

There were significant differences between pre- and post-treatment networks in network invariance (*M* = 0.24, *p* = .001) and global network strength (*S* = 0.43, *p* = .021), with post-treatment networks exhibiting greater overall connectivity (i.e., higher global strength). Four edges significantly differed between pre- and post-treatment responder networks. Compared to pre-treatment networks, post-treatment networks exhibited a stronger positive association between resistance of obsessions and resistance of compulsions, *p* < .001, interference due to compulsions and resistance of compulsions, *p* < .001, and distress due to obsessions and difficulty controlling compulsions, *p* = .043. Pre-treatment networks exhibited a stronger positive association between difficulty controlling compulsions and difficulty resisting compulsions, *p* = .028.

As differences in network invariance and global network strength were found from pre- to post-treatment in the full sample, we separately examined changes in these indices in responders and non-responders. However, for responder post-treatment networks, correlation stability coefficients were poor (.13), suggesting that network structure and strength centrality indices were uninterpretable [29; see Supplementary Figures S7 and S8]. Correlation stability coefficients were low but interpretable (0.28) for non-responder post-treatment networks (see Supplementary Figures S7 and S8). However, because responder post-treatment networks were uninterpretable, we could not examine whether changes in network invariance and global network strength differed among responders and non-responders.

## Discussion

The present study examined differences in pre-treatment OCD symptom networks among patients with OCD who were classified as responders or non-responders at post-treatment. Additionally, this was the first study to examine changes in network structure, centrality, and global network strength from pre- to post-treatment in patients with OCD. Findings revealed strengths and limitations in using network-based approaches to symptom structures for psychopathology.

Overall, time spent performing compulsions, time spent obsessing, and interference due to compulsions had the highest strength centrality in the responder network, whereas distress associated with compulsions, distress associated with obsessions, and interference due to compulsions had the highest strength centrality in the non-responder network. These results are partially consistent with Kuckertz et al. [15], who also found that interference due to compulsions and time spent performing compulsions had the highest strength centrality in responder networks, and that interference due to compulsions was among the strongest nodes in non-responder networks. However, unlike Kuckertz et al. [15], we examined whether these nodes were significantly different in strength centrality relative to the strength centrality of other nodes in the network. We found that symptoms with the highest strength centrality in both responder and non-responder networks did not significantly differ from the strength centrality of most other nodes, suggesting that symptom centrality was not meaningfully differentiated at pre-treatment within either network. In other words, the degree to which certain symptoms had greater connectivity with other symptoms was not distinguishable in both groups, implying that the symptom interconnections may have been more diffuse and less hierarchically organized at pre-treatment. This was further emphasized by our results showing no significant differences between pre-treatment responder and non-responder networks in network invariance or global strength, deviating from prior work [15]. This suggests that baseline OCD symptom network topology alone may be insufficient to discriminate responders from nonresponders when using partial correlation networks. This is similar to prior findings showing that pre-treatment network differences between treatment responders and non-responders were small, required a very large sample size to detect, and may have reflected a confound related to symptom variation (which may be limited due to categorizing groups based on treatment response) rather than indicative of a worse mental health profile [33]. Taken together, our results suggested that pre-treatment network structures may be inadequate in distinguishing between treatment responders and non-responders. Future research might expand upon our present findings by considering additional variables beyond symptom-level items (e.g., obsessive beliefs, experiential avoidance) that might more readily distinguish responders and non-responders at pre-treatment.

The network theory of psychopathology suggests that higher global network strength (stronger associations between symptoms overall) most commonly reflects higher activation (or increased levels) of one or more symptoms, causing greater activation of other symptoms. Thus, if this theory is correct, one would expect that following ERP, there would be lower global network strength. However, we found increased global network strength post-treatment (stronger associations between OCD symptoms) compared to pre-treatment, consistent with several prior network treatment studies [20–23]. This was also consistent with a prior study on network changes across treatment in OCD, which found that participants with more favorable treatment trajectories exhibited more densely connected networks at post-treatment [16]. As such, global network strength may be better conceptualized as capturing both symptom activation and symptom deactivation. That is, just as elevations in certain symptoms can activate and exacerbate other symptoms within the network, it is also possible that core low or relatively dormant symptoms may serve a protective function, buffering against overall symptom severity and contributing to network deactivation. This conceptualization aligns with the theorized maintenance cycle of OCD, wherein interrupting one component of the cycle (e.g., reduced resistance to obsessions) can slow or disrupt the progression of other components (e.g., reduced resistance to compulsions). As a result, these processes may remain strongly connected, even when their association reflects symptom attenuation rather than exacerbation.

This is further exemplified by our findings showing stronger positive associations between certain edges at post-treatment relative to pre-treatment. Specifically, compared to pre-treatment networks, post-treatment networks exhibited a stronger positive association between resistance of obsessions and resistance of compulsions, resistance of compulsions and interference due to compulsions, and distress due to obsessions and difficulty controlling compulsions. These stronger post-treatment associations might reflect the successful implementation of ERP. In particular, after treatment, participants may have had a greater ability to effectively resist engaging maladaptively with obsessional thoughts, which in turn, strongly helped their ability to resist compulsions. Similarly, the strong association between the ability to resist compulsions and interference with compulsions may indicate that the more patients resisted performing compulsions, the more their functional impairment also decreased. Moreover, the less patients were distressed by their obsessions, the easier it may have been to control compulsions. This again is consistent with the maintenance cycle for OCD, which suggests that engagement with obsessions increases distress and ultimately reinforces engagement with compulsions, and vice versa [3]. It also makes sense that fewer compulsions meant less overall functional impairment because compulsions tend to be time-consuming and result in significant disruption in daily activities.

Our findings showing a change in network structure from pre- to post- treatment were inconsistent with several prior studies on GAD, MDD, and PTSD, which showed no differences in network invariance from pre- to post- treatment [20, 21, 23]. Based in part on these prior findings, Schlesselmann et al. [25] argued that there may be limited utility in examining treatment effects using cross-sectional network analysis. However, our results refute this suggestion. One possible explanation for these discrepancies is that, unlike prior studies, the present study examined intensive, daily outpatient treatment over an average of 10 weeks, whereas in prior studies treatment lasted as little as three sessions [23], an average of 8 days [20] or 2–3 weeks [21]. Therefore, substantial changes in network structure may be discernible when the optimal treatment dose is received, and this may be indicative of lasting change and lower vulnerability to relapse. Clinically, this suggests that ERP may be associated with changes in how OCD symptoms relate to one another and that changes in symptom network structure over time may represent an important treatment outcome. Future research might benefit from incorporating session-by-session data to examine how and when network structure changes during ERP, and whether a minimum or optimal treatment dose (e.g., 10 weeks) is required for such changes to emerge.

Taken together, these findings offer important implications for treatment. The present findings appear to corroborate the importance of the OCD maintenance cycle, suggesting that post-treatment networks are characterized by strong associations between symptoms that may deactivate, rather than exacerbate, the symptom network. For example, targeting resistance to obsessions may also reduce resistance to compulsions and vice versa, contributing to a deactivation cycle of decreased symptoms. Translating this to treatment, clinicians may benefit from targeting core maintenance processes implicated in the present study, such as resistance and control, that may potentially yield broader downstream reductions in other symptoms, such as distress and functional interference, further reducing overall symptom severity. Clinicians may also benefit from more carefully considering how OCD symptoms are associated when formulating treatment. For example, for clients who struggle to resist compulsions, initially targeting resistance to obsessions may serve as an effective entry point, potentially making it easier to resist compulsions over time (as opposed to trying to target both at once, or trying to target a symptom not strongly implicated to be associated with resistance to or control of compulsions in the network, such as distress due to compulsions). Thus, careful consideration of the implications of change in the symptom networks in OCD may help refine clinical case conceptualization by highlighting shared maintaining processes that, when targeted, may yield broader treatment gains.

Another important implication of this work is that network analysis may be better suited for capturing mechanisms of therapeutic change rather than as a prognostic indicator of treatment response. As noted by Lee et al. [33], when only examining network differences at baseline as a prognostic indicator of treatment response, differences between treatment responders and non-responders are often small, require very large samples to detect, and may be confounded by lower symptom variance. Similarly, in the present study, whereas pre-treatment networks appeared relatively stable and showed little differentiation between responders and non-responders, post-treatment networks exhibited statistically significant changes in network structure and global network strength, suggesting that symptom connectivity may be more informative as a marker of change rather than a predictor of outcome. This distinction is important given the breadth of literature examining networks as pre-treatment indicators [e.g., 15, 23, 34]. A more informative approach may be to examine how specific symptom associations and overall network structures evolve across sessions to identify *when* patients begin to respond to treatment and *how* these changes correspond to shifts in symptom interrelations. Future research should incorporate session-by-session symptom data to more precisely pinpoint mechanistic shifts in symptom networks over the course of treatment.

Our study had several limitations that should be taken into consideration when interpreting our findings. Data was collected from health records from multiple treatment centers across the US, which meant that certain demographic information (including gender, race/ethnicity, and socioeconomic status) and clinical indicators (severity of comorbidity, duration of illness, age of onset, and medication use) were not available. This resulted in several limitations. First, unavailable information regarding race and ethnicity precludes our ability to generalize our findings to underrepresented communities. As the few network studies conducted to date on OCD are in primarily White, non-Latine populations, it is crucial to increase representation in future research. Additionally, clinical characteristics such as medication use and duration of illness could have influenced treatment response. Although it was not our goal in this manuscript to examine individual differences as moderators of network change in OCD symptoms, future research with larger samples and more individual difference characteristics available at baseline should examine potential clinical indicators as network moderators, such as differences in racial/ethnic identity, comorbidity, or current medication use.

Moreover, although the stability of our correlation coefficients was considered interpretable for pre-treatment comparisons and comparisons from pre-to-post treatment in the full sample, some values fell below the preferred threshold (0.5; Epskamp et al. [29]). This was likely due in part to reduced variability among participants at post-treatment, as the majority of the sample (62.6%) were treatment responders and thus exhibited less variability across symptoms within the network. For this reason, although meaningful information could have been gained from examining responders and non-responders separately at pre- and post-treatment, the limited variance at post-treatment, particularly among responders, likely resulted in the unstable and uninterpretable network estimates. Although it is plausible that this would continue to be an issue even with large sample sizes [see 33], studies with larger samples may allow for examination of responders and non-responders separately throughout treatment. Another limitation was that our analyses were based on between-person networks estimated at two time points. Consequently, we were unable to discriminate between-person and within-person symptom associations or to evaluate temporal directionality. Idiographic or multilevel approaches, which require intensive longitudinal data (e.g., session-by-session or ecological momentary assessments), would enable examination of how symptom associations changed within individuals over time. Future studies incorporating more frequent symptom assessments could better clarify the temporal sequencing of change and the dynamic interplay among symptoms during treatment.

In a sample of adults with OCD who underwent IOP ERP treatment, we found that OCD symptom networks were not significantly different between treatment responders and non-responders at pre-treatment. However, global network strength, which reflects the strength of association between symptoms of the network, was significantly different between pre- and post- treatment, with post-treatment networks exhibiting significantly greater network strength and stronger positive associations between resistance of obsessions and resistance of compulsions, resistance of compulsions and interference due to compulsions, and distress due to obsessions and difficulty controlling compulsions. This may suggest that resistance of and distress over obsessions, resistance of and control of compulsions, and/or interference from compulsions may be important targets and potential mechanisms of change that when reduced by ERP individually and/or jointly may help reduce one another. It also suggests that greater post-treatment global network strength might reflect successful implementation of ERP and deactivation of symptoms, rather than being indicative of greater symptom activation and psychological vulnerability. Aligning with prior work [33], the present study also demonstrated that network analysis may be better suited for capturing mechanisms of therapeutic change rather than discriminating responders from nonresponders using baseline symptom networks. Indeed, this was the first study to examine changes in network structure and global network strength from pre- to post-treatment in patients with OCD, revealing that both indices changed significantly across treatment. These findings suggest that ERP may be associated with changes in network structure and connectivity over the course of treatment, highlighting the potential value of examining session-by-session network dynamics in future research to pinpoint when and how these mechanistic changes occur. Given the present results, future research would benefit from further investigation of the replicability of cross-sectional networks, idiographic approaches, and the interpretability of pre–post intervention networks.

## Supporting information

10.1192/j.eurpsy.2026.10184.sm001 Swisher et al. supplementary material Swisher et al. supplementary material

## Data Availability

The data that support the findings of this study are available from the second author, K.R., upon reasonable request.

## Footnotes

1 The OCD Anxiety Centers are a group of mental health clinics in the US, providing standardized specialty treatment for OCD and anxiety disorders at the intensive outpatient level of care.

## Supplementary material

The supplementary material for this article can be found at http://doi.org/10.1192/j.eurpsy.2026.10184.
