# Supplementary material for: A network analysis of obsessive-compulsive patients in intensive outpatient treatment
Source: Eur Psychiatry. 2026 Mar 23;69(1):e46. doi: 10.1192/j.eurpsy.2026.10184 (PMC13122522; doi:10.1192/j.eurpsy.2026.10184)
Supplement: Swisher et al. supplementary material [file S0924933826101849sup001.docx]

**Figure S1.** Nonparametric bootstrapped 95% confidence intervals for edge weights of pre-treatment responder and non-responder network edges


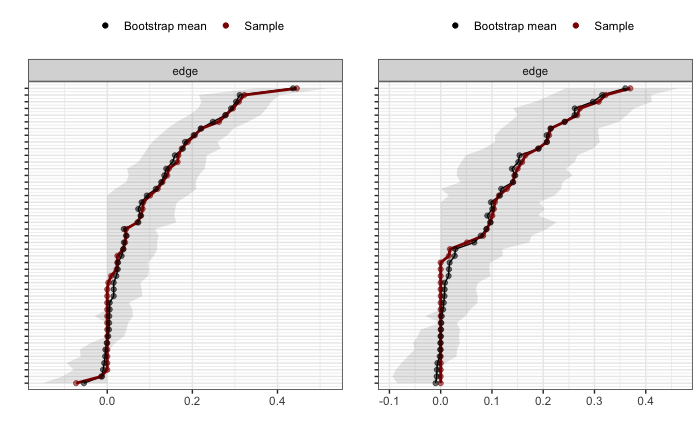


*Note*. Responders (i.e., participants who experienced ≥ 35% reduction in YBOCS scores from pre- to post-treatment) at pre-treatment are presented on the left. Non-responders at pre-treatment are on the right. The red line indicates the edge weight values and the gray area the 95% CI.

**Figure S2.** Strength centrality stability for responder and non-responder networks at pre-treatment

*
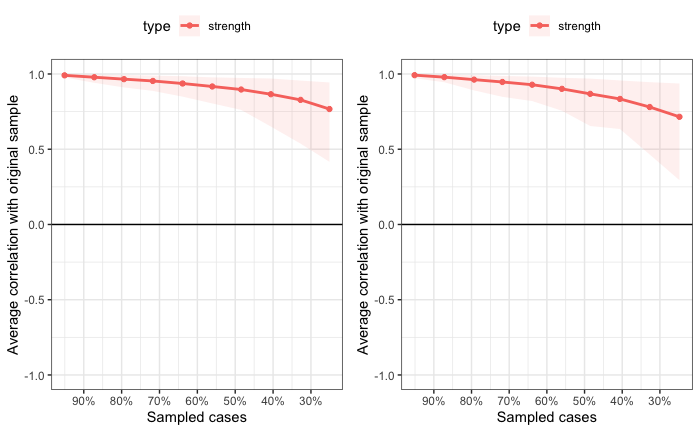
*

*Note*. Responders (i.e., participants who experienced ≥ 35% reduction in YBOCS scores from pre- to post-treatment) at pre-treatment are presented on the left. Non-responders at pre-treatment are on the right. Figure depicts stability coefficients for strength centrality when using case-dropping bootstrap.

**Figure S3**. Bootstrapped different test for strength centrality for responder and non-responder pre-treatment networks


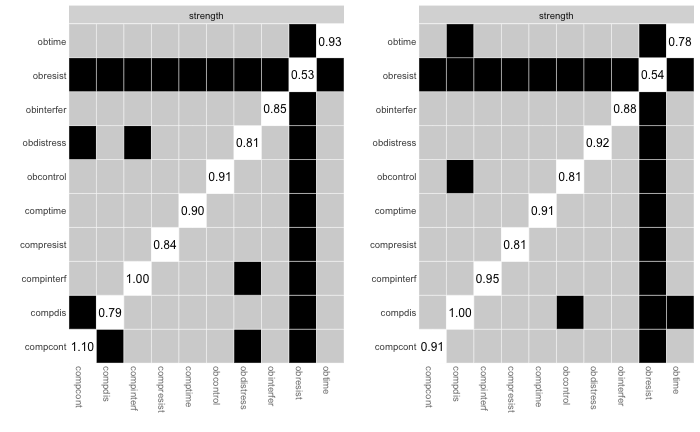


*Note.* Responders at pre-treatment bootstrapped difference test for strength are presented on the left, non-responders at pre-treatment on the right. Gray boxes indicate strength centrality that did not significantly differ from one another. Black boxes indicate strength centrality that did significantly differ. Obinterfer = interference due to obsessions; Obdistress = distress caused by obsessions, Obresist = difficulty resisting obsessions; Obcontrol = difficulty controlling obsessions; Comptime = time consumed by compulsions; Compinterf = interference due to compulsions; Compdis = distress caused by compulsions; Compresis = difficulty resisting compulsions; Compcont = difficulty controlling compulsions

**Figure S4.** Nonparametric bootstrapped 95% confidence intervals for edge weights of pre-treatment and post-treatment networks for the entire sample.


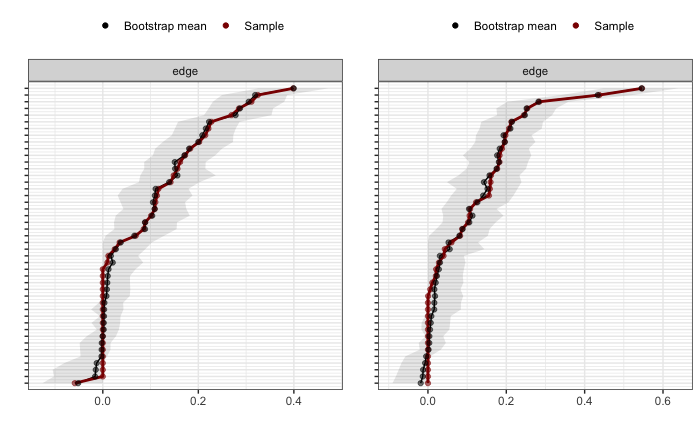


*Note*. The entire sample at pe-treatment for the entire sample is on the left and post-treatment on the right. The red line indicates the edge weight values and the gray area the 95% CIs

**Figure S5.** Strength centrality stability for pre-treatment and post-treatment networks for the entire sample


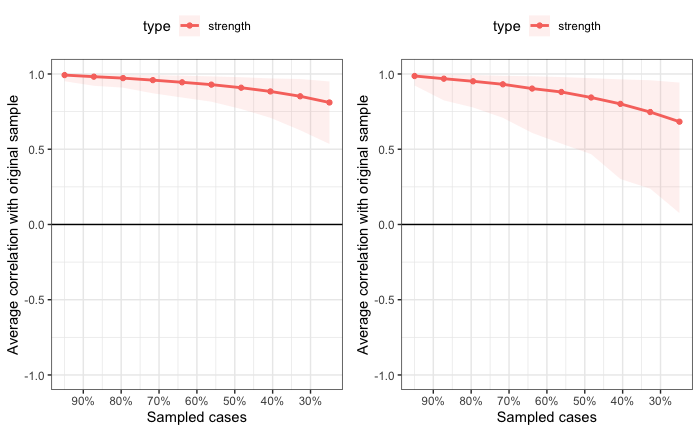


*Note*. The entire sample at pre-treatment is shown on the left and post-treatment on the right. Figures depict stability coefficients for strength centrality when using case-dropping bootstrap.

**Figure S6**. Bootstrapped different test for strength centrality for pre and post-treatment networks in the entire sample.


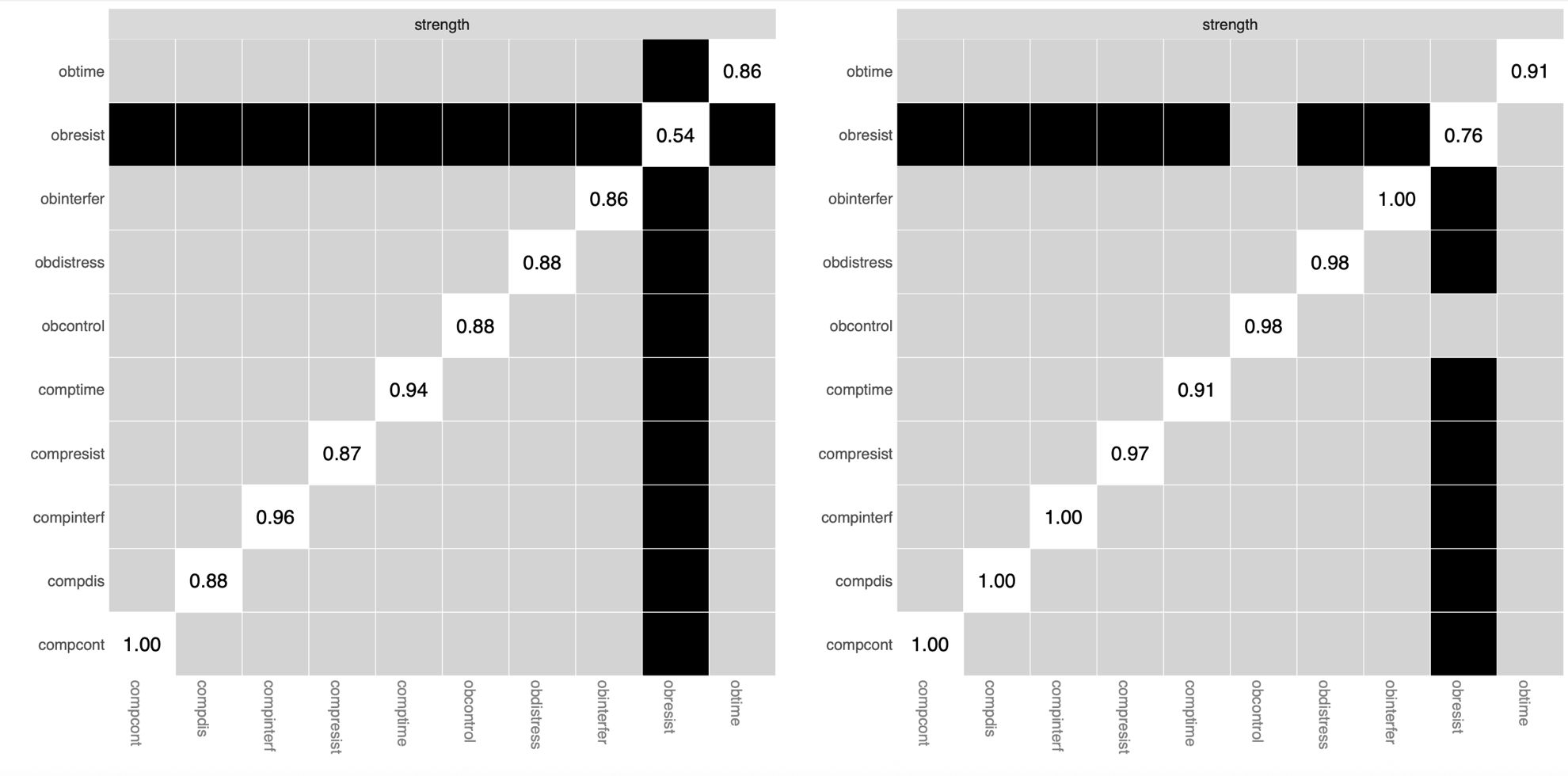


*Note.* Pre-treatment bootstrapped difference test for strength centrality for the entire sample is presented on the left, post-treatment is presented on the right. Gray boxes indicate strength centrality that did not significantly differ from one another. Black boxes indicate strength centrality that did significantly differ. Obinterfer = interference due to obsessions; Obdistress = distress caused by obsessions, Obresist = difficulty resisting obsessions; Obcontrol = difficulty controlling obsessions; Comptime = time consumed by compulsions; Compinterf = interference due to compulsions; Compdis = distress caused by compulsions; Compresis = difficulty resisting compulsions; Compcont = difficulty controlling compulsions

**Figure S7.** Nonparametric bootstrapped 95% confidence intervals for edge weights of post-treatment responder and non-responder network edges

**
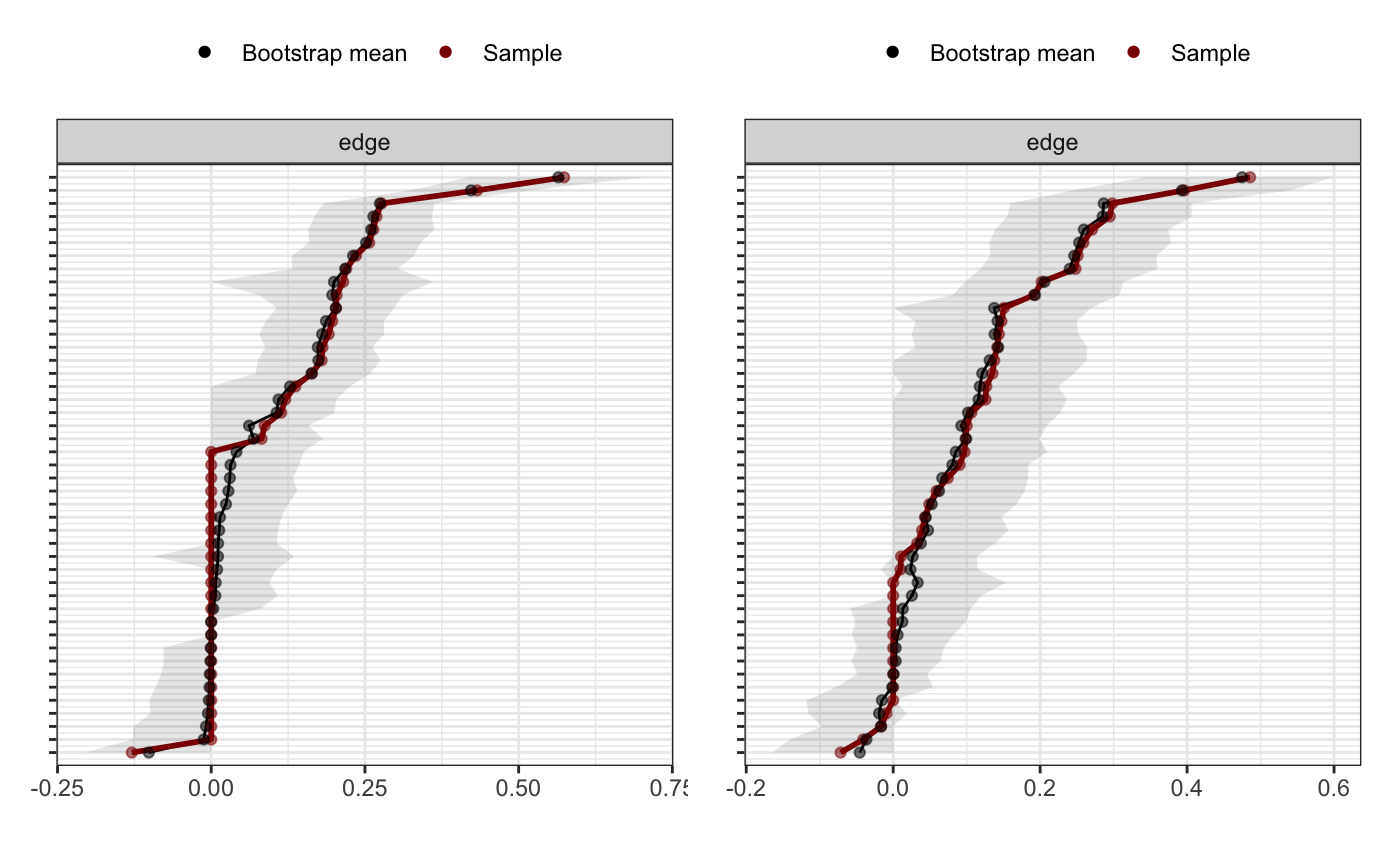
**

*Note.* Responders at post-treatment are on the left. Non-responders at post-treatment are on the right. The red line indicates the edge weight values and the gray area the 95% C

**Figure S8.** Strength centrality stability for post-treatment responder and non-responder networks


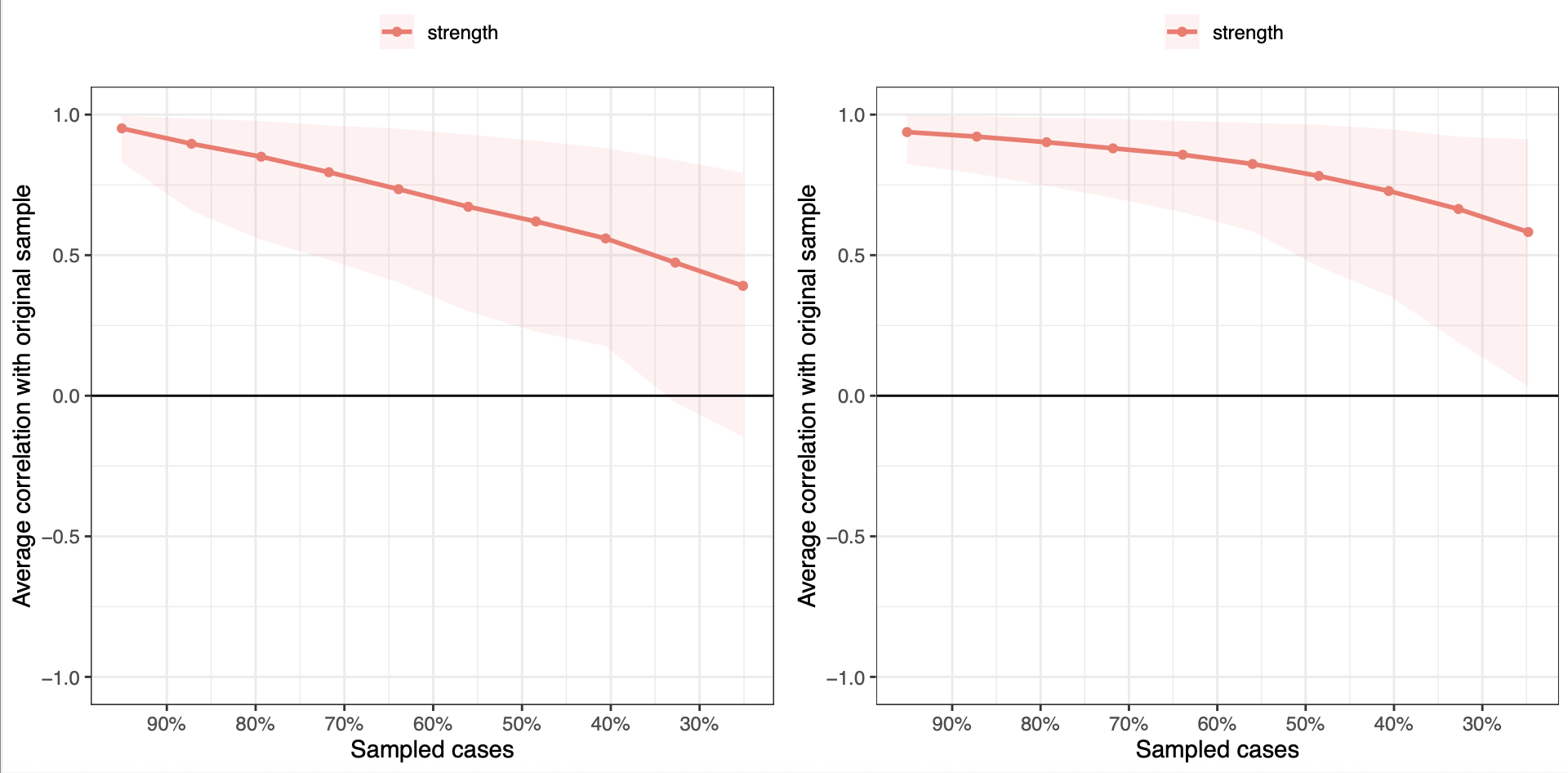


*Note*. Responders at post-treatment are shown on the left and non-responders at post-treatment on the right. Figures depict stability coefficients for strength centrality when using case-dropping bootstrap.
